# Cigarette and waterpipe smoking among adolescents in Estonia: HBSC survey results, 1994–2006

**DOI:** 10.1186/1471-2458-8-392

**Published:** 2008-11-25

**Authors:** Kersti Pärna, Janika Usin, Inge Ringmets

**Affiliations:** 1 Department of Public Health, University of Tartu, Estonia; 2 Estonian Centre of Excellence in Behavioural and Health Sciences, Tallinn, Tartu, Estonia

## Abstract

**Background:**

Smoking is a major single cause of preventable morbidity and premature mortality. Tobacco use among adolescents is a significant public health problem as smoking behaviour is undeniably established in adolescence. While cigarette smoking among adolescents has been a significant public health problem for years, waterpipe smoking is considered to be a new global public health threat. The objectives of this study were to describe trends of cigarette smoking and the prevalence of waterpipe smoking and to study the association between cigarette and waterpipe smoking among adolescents in Estonia.

**Methods:**

This study was based on a four-yearly HBSC survey of health behaviour among school-aged children conducted in 1994–2006 in Estonia. It was a school-based survey of a nationally representative sample using standardized methodology. The target group of the survey were 11-, 13-, and 15-year-old schoolchildren (N = 13826), 6656 boys and 7170 girls. Cigarette and waterpipe smoking was determined on a 4-stage scale: every day, at least once a week, less than once a week, not smoking. Logistic regression analysis was applied to examine gender- and age-specific smoking trends and to study the association between cigarette and waterpipe smoking.

**Results:**

Prevalence of smoking was higher among boys than girls in all age groups during the whole study period. The prevalence of cigarette smoking increased in 1994–2002 and then slightly decreased in both genders. The increase in smoking was larger among girls. Among girls, daily smoking increased during the whole study period. Among 15-year-old schoolchildren one-third of the boys and one quarter of the girls were cigarette smokers, 21% of the boys and 12% of the girls were daily smokers in 2006. One fourth of the boys and one sixth of the girls were waterpipe smokers. A logistic regression analysis revealed a strong association between cigarette and waterpipe smoking among schoolchildren.

**Conclusion:**

The results of this study can significantly enhance the capacity to develop and implement tobacco prevention and control programmes among the youth in Estonia.

## Background

Since the collapse of the Soviet Union in 1991, Estonia, one of the Baltic countries, has experienced major political, economic and social changes. This transition was accompanied by an immediate increase in mortality, which only reversed in 1995 [1,2]. Also, the gross national product per capita started to rise again from the mid-1990s. In 2004, Estonia became a member of the European Union associated with introduction of the single European market and the general pressure towards convergence in many policy areas.

Smoking is a major single cause of preventable morbidity and premature mortality [3]. Although the vast majority of smoking-related deaths occur in middle-aged and elderly people, smoking behaviour is undeniably established in adolescence [4,5]. Moreover, individuals who begin smoking at a younger age have an increased risk of becoming daily smokers [6]. Therefore, data about the prevalence of tobacco use and smoking trends of a country are urgently needed to make decisions about future smoking prevention policies.

While cigarette smoking among adolescents has been a significant public health problem for many years [4,7-9], waterpipe smoking is considered to be a new global public health threat [10]. Waterpipes have been used to smoke tobacco and other substances in Africa and Asia for at least four centuries [11]. Since the 1990s waterpipe smoking appears to have been spreading among new populations, such as young people in European countries (including Estonia, one of the Baltic countries). Commercial marketing may also contribute to the spread of waterpipe smoking across the globe. At the same time data on the prevalence of waterpipe smoking in western countries is limited [12]. Many people believe that waterpipe smoking is safer than cigarette smoking because the smoke is filtered through water [11]. Contrary to popular belief, the smoke that emerges from a waterpipe contains numerous toxicants known to cause lung cancer, heart diseases, and other diseases similarly to cigarette smoking [13-15]. Waterpipe tobacco smoking delivers the addictive drug nicotine, and, similarly to other tobacco products, more frequent use leads to addiction [15,16]. Nicotine intakes appear to be even higher than from cigarette smokers, which may explain the emerging evidence of dependence [12]. Waterpipe smoking is often social, and two or more people may share the same waterpipe [13]. All these findings reinforce the need to conduct research on waterpipe smoking in order to have more evidence-based information.

The objectives of the present article were to (1) describe trends in cigarette smoking in 1994–2006, (2) study the prevalence of waterpipe smoking in the last study year, and (3) study the association between cigarette and waterpipe smoking among adolescents in Estonia.

## Methods

The present paper is based on the Estonian part of the Health Behaviour in School-aged Children (HBSC) survey, which is a WHO collaborative cross-national study. The aim of the HBSC survey is to describe health and health behaviour of adolescents and its relationship to the social context.

The HBSC study consists of cross-sectional surveys based on an internationally agreed protocol and is carried out among country-representative samples of 11-, 13- and 15-year-old schoolchildren every fourth year. The latest survey in 2006 covered in total more than 40 countries. A detailed description of the aims and the theoretical framework of the study can be found elsewhere [17].

### Sample

HBSC is a school-based study, and all four surveys since 1994 in Estonia used identical protocols with regard to the target group. The target population of the survey were 11-, 13- and 15-year-old schoolchildren. All comprehensive schools in Estonia were eligible for the study. Technically the sampling procedure adhered to the following scheme. Estonia was divided into four major geographic regions. Each region was stratified by urbanisation and study language in the school. As the next step, random selection of the schools in the strata was performed. The probability of schools being selected was proportional to the number of schoolchildren enrolled in the specified grades in the strata. The recommended minimum sample size for each of the three age groups was set internationally. In the schools, cluster sampling was used where primary sampling unit was the class. All schoolchildren in selected classes attending school on the day of the survey were eligible to participate. Fieldwork for each cross-national survey was carried out over a period of around seven to eight months, from October to May of the following year. This reflected the sampling strategy to achieve the mean ages of 11.5, 13.5 and 15.5. In International Data Bank at the Norwegian Social Science Data Services the collected data were checked and cleaned. Data for young people outside the targeted age ranges were removed [18].

### Data collection

This survey was approved by Ethics Review Committee on Human Research of the University of Tartu, Estonia (2005, protocol 142/98). Headmasters of the schools and representatives of the parents were notified by a letter in advance of the survey and schoolchildren gave verbal consent to complete the questionnaire. Schoolchildren participated in the survey on a voluntary basis.

Data was collected through self-completion standardized questionnaires administered by teachers or members of the research group in the classroom. After completion the students were asked to put the questionnaire in an envelope, seal it, and hand it over to the teacher or a member of the research group. The study was totally anonymous, and thus it was impossible to conduct an individual non-response analysis.

### Variables

Cigarette and waterpipe status were defined on the basis of the question 'How often do you smoke tobacco at present?' Possible responses included 'every day', 'at least once a week, but not every day', less than once a week', or 'never'. Adolescents who smoked 'less than once a week' or more often were considered to be cigarette smokers. Cigarette smoking was analysed for the whole study period. The question concerning waterpipe smoking was added to the questionnaire in 2006 and could consequently only be examined in the final year of the study.

The age of starting smoking was based on the question 'At which age did you smoke the first cigarette?' The question was added to the questionnaire before the survey in 2002. In 2002 it was an open-ended question, but in 2006 it became a multiple-choice question with the following possible responses: at the age of 11, 12, 13, 14, 15, 16 years.

### Statistical analysis

The data were analysed using the statistical package Stata 9. Analysis was done separately for boys and girls. Time trends for the percentages of cigarette and the prevalence of waterpipe smoking were described by using frequency tables. Mean age of starting smoking and standard deviation (SD) were calculated for boys and girls. The Student's t-test was used to compare the age of starting smoking between the genders (significance level 0.05). Time trends of cigarette smoking and prevalence of waterpipe smoking were analysed for each age group: 11-, 13- and 15-year-olds. An additional analysis focused on the gender ratio in smoking (smoking prevalence of boys/smoking prevalence of girls) calculated for each survey period.

Time trends of cigarette smoking were estimated by logistic regression models in each age group. Cigarette smoking was included in the model as a dependent variable and the survey year as an explanatory variable. The association between cigarette and waterpipe smoking was explored by logistic regression analysis where cigarette smoking was again a dependent variable and waterpipe smoking (occasional and at least weekly smoker, non-smoker) as an explanatory variable. The results of logistic regression analysis were presented as odds ratios (OR) and 95% confidence intervals (CI). The association between cigarette and waterpipe smoking was controlled for age.

Data concerning age group was missing from 10 and data concerning smoking status from 40 completed questionnaires. These incomplete questionnaires were omitted from the analysis. A total of 13826 questionnaires (6656 boys and 7170 girls) were used in this study.

## Results

### Cigarette smoking

Tables 1, 2, 3 show the cigarette smoking prevalence classified by survey year among 11-, 13- and 15-year-old boys and girls. In all age groups, the prevalence of cigarette smoking increased from 1994 to 2002, followed by stabilization in 2006. The prevalence of cigarette smoking was higher among boys compared to girls in all age groups during the whole study period.

**Table 1:** Cigarette smoking among 11-year-old adolescents, HBSC survey 1994–2006

Cigarette smoking	1994		1998		2002		2006	
	
	N	%	N	%	N	%	N	%
	Boys							
Smokers	21	3.9	14	6.2	49	7.3	31	4.6
Every day	2	0.4	0	0.0	10	1.5	2	0.3
At least once a week	2	0.4	3	1.3	15	2.2	10	1.5
Less than once a week	17	3.1	11	4.9	24	3.6	19	2.8
Non-smokers	524	96.2	213	93.8	624	92.7	653	95.5

Total	545	100	227	100	673	100	684	100

	Girls							
Smokers	3	0.5	4	1.6	17	2.8	12	1.7
Every day	0	0.0	1	0.4	2	0.3	1	0.1
At least once a week	1	0.2	1	0.4	7	1.2	3	0.5
Less than once a week	2	0.3	2	0.8	8	1.3	8	1.1
Non-smokers	619	99.5	246	98.4	593	97.2	720	98.4

Total	622	100	250	100	610	100	732	100

**Table 2:** Cigarette smoking among 13-year-old adolescents, HBSC survey 1994–2006

Cigarette smoking	1994		1998		2002		2006	
	
	N	%	N	%	N	%	N	%
	Boys							
Smokers	55	10.4	53	14.4	139	20.2	113	15.6
Every day	8	1.5	16	4.3	52	7.6	47	6.5
≥ 1× a week	21	4.0	11	3.0	36	5.2	32	4.4
<1× a week	26	4.9	26	7.1	51	7.4	34	4.7
Non-smokers	476	89.6	316	85.6	548	79.8	612	84.4

Total	531	100	369	100	687	100	725	100

	Girls							
Smokers	18	2.8	19	4.2	112	15.3	84	11.3
Every day	3	0.5	3	0.7	29	4.0	30	4.1
≥ 1× a week	4	0.6	4	0.9	30	4.1	23	3.1
<1× a week	11	1.7	12	2.6	53	7.2	31	4.2
Non-smokers	615	97.2	437	95.8	622	84.7	655	88.6

Total	633	100	456	100	734	100	739	100

**Table 3:** Cigarette smoking among 15-year-old adolescents, HBSC survey 1994–2006

Cigarette smoking	1994		1998		2002		2006	
	
	N	%	N	%	N	%	N	%
	Boys							
Smokers	160	29.3	78	31.1	229	37.0	260	32.5
Every day	85	15.6	43	17.1	144	23.3	168	21.0
≥ 1× a week	33	6.0	17	6.8	44	7.1	44	5.5
<1× a week	42	7.7	18	7.2	41	6.6	48	6.0
Non-smokers	386	70.7	173	68.9	390	63.0	539	67.5

Total	546	100	251	100	619	100	799	100

	Girls							
Smokers	59	9.5	63	18.9	165	25.5	198	25.2
Every day	20	3.2	26	7.8	75	11.6	92	11.7
≥ 1× a week	16	2.6	14	4.2	43	6.6	54	6.9
<1× a week	23	3.7	23	6.9	47	7.3	52	6.6
Non-smokers	569	90.6	271	81.1	483	74.5	586	74.7

Total	628	100	334	100	648	100	784	100

Among 11-year-old schoolchildren smoking increased from 1994 to 2002, followed by stabilization (Table 1). While in 1994 the prevalence of smoking was 3.9% among boys and 0.5% among girls, in 2002 the respective prevalence rates were 7.3% and 2.8%.

Among 13-year-old boys and girls the time trend of cigarette smoking was similar to the younger age group (Table 2). While in 1994 the prevalence of smoking among boys was 10.4%, in 2002 it was nearly twice as high (20.2%). At the same time smoking among 13-year-old girls increased over five times from 2.8% in 1994 to 15.3% in 2002. The biggest difference in the prevalence of smoking between boys and girls was in the first study year and the smallest in the last study year (gender ratios 3.7 and 1.4, respectively). Among boys, stabilization of daily smoking prevalence occurred after an increase between 1994 and 2002. Among girls the prevalence of daily smoking increased during the whole study period from 0.5% to 4.1%. The gender ratio of daily smoking was the smallest (1.6) in the last study year.

Among 15-year-olds the prevalence of cigarette smoking increased from 1994 to 2002; among boys it increased from 29.3% to 37.0% and then slightly decreased to 32.5% (Table 3). The prevalence of daily smoking among boys followed the same trend being the highest in 2002 (37.0%). By comparison with boys, the increase of smoking among girls was remarkably higher (from 9.5% to 25.5%) during 1994–2002 followed by stabilization in 2006. The prevalence of daily smoking among girls increased during the whole study period from 3.2% to 11.7%. During the study period the difference in smoking prevalence between boys and girls decreased (gender ratios 3.1 and 1.3, respectively). The gender difference in daily smoking followed the same trend (gender ratio was 4.9 in 2002 and 1.8 in 2006).

In 2002, the odds to smoke was 1.96 times higher than in the first study year among 11-year-old boys (among 13-year-olds 2.20 and 15-year-olds 1.42) (Table 4). The situation among girls was different. Compared to the first study year, the odds ratio to smoke was statistically significantly higher in each age group in the other study years. Compared to the first study year, the highest odds ratio to smoke was among 11- and 13-year-old girls in 2002 (5.92 and 6.15, respectively).

**Table 4:** Prevalence odds ratio (POR) for cigarette smoking and 95% confidence interval (CI) among 11–15-year-old adolescents by study year, HBSC survey 1994–2006

Study year	POR (95% CI)		
	
	Age groups		
	11-year-olds	13-year-olds	15-year-olds
	
	Boys		
1994	1	1	1
1998	1.64 (0.82–3.29)	1.45 (0.97–2.17)	1.09 (0.79–1.51)
2002	1.96 (1.16–3.31)	2.20 (1.57–3.07)	1.42 (1.11–1.81)
2006	1.19 (0.67–2.09)	1.60 (1.13–2.25)	1.16 (0.92–1.47)

	Girls		
1994	1	1	1
1998	3.36 (0.75–15.10)	1.49 (0.77–2.86)	2.24 (1.53–3.29)
2002	5.92 (1.72–20.29)	6.15 (3.69–10.25)	3.30 (2.39–4.54)
2006	3.44 (0.97–12.24)	4.38 (2.60–7.38)	3.26 (2.38–4.46)

In 2002 the mean age of starting smoking was 10.0 (SD ± 2.5) among boys and 11.7 (SD ± 2.2) among girls. In 2006 the respective ages were 11.8 (SD ± 1.1) and 12.3 (SD ± 1.3). In both study years girls started to smoke significantly later than boys (p < 0.001).

### Waterpipe smoking

In 2006, the prevalence of waterpipe smoking was 25.2% among boys and 16.2% among girls. Waterpipe smoking increased with age (Table 5). Among 11-year-old boys the prevalence of waterpipe smoking was 10.0%; among girls it was 2.9%, among 13-year-olds 25.1% and 13.3%, among 15-year-olds 38.1%, and 31.4%, respectively. The gender difference in waterpipe smoking was smallest among 15-year-old adolescents.

**Table 5:** Prevalence of waterpipe smoking among 11–15-year-old adolescents, HBSC survey 2006

Waterpipe smoking	Age group					
	11-year-olds		13-year-olds		15-year-olds	
	
	N	%	N	%	N	%
	Boys					
Every day	3	0.4	10	1.4	15	1.9
≥ 1× a week	9	1.3	32	4.4	61	7.6
<1× a week	57	8.3	140	19.3	230	28.8
Do not smoke	593	86.7	520	71.7	482	60.3
Missing answer	22	3.2	23	3.2	11	1.4

Total	684	100	725	100	799	100

	Girls					
Every day	1	0.1	2	0.3	3	0.4
≥ 1× a week	5	0.7	18	2.4	25	3.2
<1× a week	15	2.1	78	10.6	218	27.8
Do not smoke	703	96.0	627	84.8	535	68.2
Missing answer	8	1.1	14	1.9	3	0.4

Total	732	100	739	100	784	100

### Association between cigarette and waterpipe smoking

The odds to smoke cigarettes was significantly higher among waterpipe smoking boys and girls compared to waterpipe non-smoking adolescents. The odds to smoke cigarettes was even 12.33 higher among those boys who smoked the waterpipe at least weekly compared to waterpipe non-smoking boys (Table 6).

**Table 6:** Prevalence odds ratio (POR) and 95% confidence interval (CI) for cigarette smoking among 11–15-year-old adolescents by waterpipe smoking, HBSC survey 2006

Waterpipe smoking	Boys	Girls
	
	OR* (95% CI)	OR* (95% CI)
Do not smoke	1	1
Less than once a week	5.82 (4.45–7.61)	4.93 (3.66–6.64)
At least once a week ((including every day)	12.33 (8.19–18.55)	4.66 (2.55–8.54)

*Adjusted for age.

## Discussion

This study was part of the international HBSC survey and concentrated on cigarette and waterpipe smoking among 11-, 13-, and 15-year-old adolescents in Estonia.

All types of smoking were described in this study. Smoking among adolescents may well show some important fluctuations in regularity, from occasional and weekly to daily smoking. Daily smoking adolescents are more likely to smoke in the future and to develop smoking-related health problems leading to premature deaths [4]. At the same time, when daily smoking is increasing, the behaviour can be overtaken by weekly and occasional smokers as main risk group of daily smoking.

The results showed that the prevalence of cigarette smoking was higher among boys than in girls in all age groups during the whole study period. At the same time, the difference in smoking prevalence between boys and girls decreased in Estonia during the study period. Boys have reported higher smoking prevalence in all of the countries from Central and Eastern Europe by HBSC [19]. Among neighbouring countries, 15-year-old boys smoking prevalence was significantly higher than girls smoking prevalence in Latvia, Lithuania, and Russia [20]. From 1994 to 2002 cigarette smoking among 11–15-year-olds increased in Estonia, followed by stabilization in the last survey year. The prevalence of daily smoking among boys followed the same trend. As a notable exception daily smoking among girls increased during the whole study period. The main increase in the rate of smoking took place in the 1990s. The same results were found in Germany [21]. In Estonia, the increasing trend of smoking among adolescents in the mid-1990s could be explained with the unfortunate consequences of the transition period consisting of the increased willingness of many young people (especially girls) to experiment with risk behaviour.

According to Hublet et al [4], three different trends were observed in daily smoking among adolescents in the HBSC survey during 1990–2002. Among boys, the Nordic countries showed a declining or stabilizing smoking trend; in the Western countries an initial increase was followed by a decrease; and in eastern European countries an increase was followed by a stabilization in smoking prevalence during 1998–2002. Girls, similarly to boys, revealed similar trends with a few exceptions. No country showed continuous decline in daily smoking in 1990–2002. Some countries showed only an increasing trend in girls while in boys some stabilization was observed. Hungary was the only country where smoking has increased since the last survey year in 2002. Thus, Estonia belongs to the eastern European countries where daily smoking was stabilized among boys but continues to increase among girls.

According to worldwide literature, boys started to smoke at a younger age than girls [18]. The present study contributed to this finding. The age difference in initiating smoking between the genders was about two years in Estonia. The age of the first cigarette clearly shows that legislation that attempts to control cigarette availability is either not fully enforced or relatively ineffective. Unfortunately, the data of the HBSC survey did not allow describing trends in initiating of smoking as the question concerning the age when one starts smoking was added to the questionnaire only in 2002. Moreover, the assessment of the age of the initiation of cigarette smoking among adolescents suffered from an obvious methodological shortcoming [18]. In 2002 it was an open-ended question, but in 2006 there was a possibility to choose the right answer from multiple choices and unfortunately the possible youngest age was 11 years. According to the previous study in Estonia, some adolescent smokers had started smoking before the age of 11 years [22]. Thus, the age of the initiation of cigarette smoking can be overestimated in the HBSC survey in 2006 and requires carefulness in making conclusions.

The results of this study showed that one quarter of the boys and one sixth of the girls were waterpipe smokers. Among 15-year-old adolescents the prevalence of less than weekly waterpipe smokers was about the same for boys and girls. This confirmed the report by WHO [13] that in some countries where cigarette smoking is more common among men, waterpipe smoking appears to be more evenly distributed between both genders. Unfortunately, it was not possible to compare waterpipe smoking in Estonia with other countries as the international data of the HBSC 2006 study were unavailable at the time of writing this paper. Although the data on the spread of the waterpipe use are scarce, the majority of the available data are from the Middle East and reveal a worrisome picture. Compared to the Middle Eastern countries, the prevalence of waterpipe smoking was lower among adolescents in Estonia. For example, among the university students of Beirut 30.6% of men and 23.4% of women reported current weekly waterpipe use in 2001. Across several countries of the Eastern Mediterranean Region, about 10–18% of the 13–15 year-olds used tobacco products other than cigarettes. In Israel 22% of the children of 12–18 years of age reported using the waterpipe at least every weekend. There is a growing recognition, with a minimal research base, that tobacco smoking using a waterpipe is increasingly common. In Estonia, the question concerning waterpipe smoking was added to the questionnaire before the latest survey in 2006. Thus, there was no data about trends in waterpipe smoking in Estonia.

This study revealed a strong association between cigarette and waterpipe smoking. According to Ward et al [23], adult waterpipe smokers showed twice higher odds of starting to smoke cigarettes compared to non-smokers of the waterpipe.

### Limitations of the study

In general, self-reported smoking prevalence has been considered to be a good indicator of the actual smoking status in epidemiology [15]. Nevertheless, the possibility of information bias cannot be excluded in large-scale school-based studies. Some students may not provide valid answers and may also underreport the level of smoking since the data collected are based on a self-administered questionnaire. The respondents were, however, assured that their answers were anonymous and that their parents and teachers would not see their answers. In addition, the importance of giving honest answers was highlighted during the administration of the questionnaires. Another limitation of the school-based study is the fact that school dropouts, which may constitute a high-risk group for smoking, were not included in the survey. Also, information referring to smokeless tobacco is lacking in this survey, but adolescents, especially boys, often use snuff. According to the Global Youth Tobacco Survey 3.3% of 13–15 year old boys and 1.4% of girls use snuff or chewing tobacco in Estonia [22]. Finally, the nature of the cross-sectional study does not allow causal inferences to be made with any confidence. Therefore, it was not possible to conclude, have cigarette smokers higher risk for waterpipe smoking or have waterpipe smokers higher risk for cigarette smoking in this survey.

Despite some limitations, this study allows systematic monitoring of tobacco use among the youth, which is the first step in the planning of prevention strategies. The results of this study raise some important policy implications for the development of cigarette smoking prevention programmes for the youth in Estonia. Moreover, the common international methodology allows further comparison of Estonia with other countries.

## Conclusion

The results of this study showed that the prevalence of smoking was higher among boys than girls in all age groups during the whole study period in Estonia, but differences in the smoking prevalence between genders decreased. The prevalence of cigarette smoking increased in 1994–2002 and then slightly decreased in both genders. The girls, but not the boys, showed an increase in daily smoking during the whole study period. Among 15-year-old adolescents, one third of the boys and one quarter of the girls were cigarette smokers, but waterpipe smokers constituted about one third. An important finding of this study was that waterpipe smoking was strongly associated with cigarette smoking. The health policy related to waterpipe smoking has lagged behind the tobacco control policy of cigarette smoking in Estonia. Despite the fact that data on use patterns, attitudes, and health risks associated with waterpipe use are especially crucial, more analytical research in this field is needed.

The results of this study can significantly enhance the capacity to develop and implement tobacco prevention and control programmes among the youth in Estonia.

## Competing interests

The authors declare that they have no competing interests.

## Authors' contributions

KP: made a substantial contribution to the conception and the design of the study, interpretation of the data, drafted the manuscript and has been involved in revising the manuscript critically. JU: participated in the design of the study, made a substantial contribution to the analysis and interpretation of the data and has been involved in revising manuscript critically. IR: participated in the design of the study, performed statistical analyses, has been involved in the interpretation of the data and in revising the manuscript critically.

## Pre-publication history

The pre-publication history for this paper can be accessed here:



## References

[B1] Leinsalu M, Vågerö D, Kunst AE (2004). Increasing ethnic differences in mortality in Estonia after the collapse of the Soviet Union. J Epidemiol Community Health.

[B2] Pärna K, Lang K, Raju K, Väli M, McKee M (2007). A rapid situation assessment of the market for surrogate and illegal alcohols in Tallinn, Estonia. Int J Public Health.

[B3] World Health Organization (1999). Gender and health in adolescence. WHO Policy Series "Health Policy for children and adolescents". International report.

[B4] Hublet A, De Bacquer D, Valimaa R, Godeau E, Schmid H, Rahav G, Maes L (2006). Smoking trends among adolescents from 1990 to 2002 in ten European countries and Canada. BMC Public Health.

[B5] Holmen TL, Barrett-Connor E, Holmen J, Bjermer L (2000). Health problems in teenage daily smokers versus nonsmokers, Norway, 1995–1997: the Nord-Trondelag Health Study. Am J Epidemiol.

[B6] Zhu BP, Liu M, Shelton D, Liu S, Giovino GA (1996). Cigarette smoking and its risk factors among elementary school students in Beijing. Am J Public Health.

[B7] World Health Organization (2007). The European tobacco control report 2007.

[B8] World Health Organization (1999). Health 21. The health for all policy framework for the WHO European Region. European health for all series No 6.

[B9] Holm K, Kremers SPJ, de Vries H (2003). Why do Danish adolescents take up smoking?. Eur J Public Health.

[B10] Chaouachi K (2006). A critique of the WHO TobReg's "Advisory Note" report entitled: "Waterpipe tobacco smoking: health effects, research needs and recommended actions by regulators". J Negat Results Biomed.

[B11] Ward KD, Weg MW Vander, Relyea G, Debon M, Klesges RC (2006). Waterpipe smoking among American military recruits. Prev Med.

[B12] Jackson D, Aveyard P (2008). Waterpipe smoking in students: prevalence, risk factors, symptoms of addiction, and smoke intake. Evidence from one British university. BMC Public Health.

[B13] World Health Organization (2005). Waterpipe tobacco smoking: heatlh effects, research needs and recommended actions by regulators. Tobacco regulation advisory note.

[B14] Maziak W, Ward KD, Afifi Soweid RA, Eissenberg T (2005). Standardizing questionnaire items for the assessment of waterpipe tobacco use in epidemiological studies. Public Health.

[B15] Maziak W, Eissenberg T, Ward KD (2005). Patterns of waterpipe use and dependence: implications for intervention development. Pharmacol Biochem Behav.

[B16] Maziak W, Ward KD, Eissenberg T (2004). Factors related to frequency of narghile (waterpipe) use: the first insights on tobacco dependence in narghile users. Drug Alcohol Depend.

[B17] Health Behaviour among School-aged children survey. (Home page). http://www.hbsc.org/.

[B18] World Health Organization (2004). Young people's health in context. Health Behaviour in School-aged Children (HBSC) study: from the 2001/2002 survey.

[B19] World Health Organization (2000). Health and health behaviour among young people.

[B20] World Health Organization (2008). Inequalities in young people's health. Health behaviour in school-aged children. International report from the 2005/2006 survey.

[B21] Richter M, Leppin A (2007). Trends in socio-economic differences in tobacco smoking among German schoolchildren, 1994–2002. Eur J Public Health.

[B22] Global Youth Tobacco Survey (GYTS). Estonian country report. http://www2.tai.ee/uuringud/Tubakas/Est_report.pdf.

[B23] Ward KD, Weg MW Vander, Relyea G, Debon M, Klesges RC (2006). Waterpipe smoking among American military recruits. Prev Med.

